# Mental Health Response in Haiti in the Aftermath of the 2010 Earthquake: A Case Study for Building Long-Term Solutions

**DOI:** 10.3109/10673229.2012.652877

**Published:** 2012-02-15

**Authors:** Giuseppe Raviola, Eddy Eustache, Catherine Oswald, Gary S Belkin

**Affiliations:** 1 From the Program in Global Mental Health and Social Change, Department of Global Health and Social Medicine, Harvard Medical School, and Department of Psychiatry, Children's Hospital Boston, Boston, MA; 2 Zanmi Lasante/Partners in Health, Central Plateau, Haiti; 3 Program in Global Mental Health and Department of Psychiatry, New York University School of Medicine

**Keywords:** capacity building, disaster, global mental health, Haiti, psychiatry, public health

## Abstract

Significant challenges exist in providing safe, effective, and culturally sound mental health and psychosocial services when an unforeseen disaster strikes in a low-resource setting. We present here a case study describing the experience of a transnational team in expanding mental health and psychosocial services delivered by two health care organizations, one local (Zanmi Lasante) and one international (Partners in Health), acting collaboratively as part of the emergency response to the 2010 Haiti earthquake. In the year and a half following the earthquake, Zanmi Lasante and Partners in Health provided 20,000 documented individual and group appointments for mental health and psychosocial needs. During the delivery of disaster response services, the collaboration led to the development of a model to guide the expansion and scaling up of community-based mental health services in the Zanmi Lasante health care system over the long-term, with potential for broader scale-up in Haiti. This model identifies key skill packages and implementation rules for developing evidence-based pathways and algorithms for treating common mental disorders. Throughout the collaboration, efforts were made to coordinate planning with multiple organizations interested in supporting the development of mental health programs following the disaster, including national governmental bodies, nongovernmental organizations, universities, foreign academic medical centers, and corporations. The collaborative interventions are framed here in terms of four overarching categories of action: direct service delivery, research, training, and advocacy. This case study exemplifies the role of psychiatrists working in low-resource settings as public health program implementers and as members of multidisciplinary teams. (Harv Rev Psychiatry 2012;20:68–77.)

In January 2010, a major earthquake struck Haiti, destroying the city of Port-au-Prince and causing massive casualties. Prior to the earthquake, Haiti was the poorest nation in the Western hemisphere,^1^ suffering from a complex situation characterized by high levels of rural and urban poverty, weak governance structures, organized crime, sporadic outbreaks of violence, and alarming levels of environmental degradation.^2^ Haiti has also had an especially deleterious history of foreign exploitation, internal division by class, and pronounced social inequities. In the context of long-standing social, political, and economic conditions that continue to foment lethal health crises such as the ongoing cholera epidemic, the earthquake emergency amounts to what has been termed an *acute-on-chronic* disaster.^3^

Scaling up of mental health services that are safe, effective, and culturally sound presents significant challenges in a situation of dramatic human loss, precipitous social change, ongoing poverty, and multiple competing needs. We present here a case study that describes the experience of a team of Haitian and American nationals working for an international health care organization, Partners in Health (PIH), in mounting a response to the earthquake focused on mental health. Historically, the problem with such acute responses is that they tend to be carried out with insufficient attention to key components necessary for sustainable delivery of care in low-resource settings. Recognizing the significant absence of formal mental health services in Haiti prior to the earthquake, PIH sought to address community mental health needs through an approach that both responded to the earthquake emergency and purposefully established a foundation for greater local capacity to address mental disorders over the long term. An intervention model, here called *5*×*5*, was developed to guide the planning of clinical services within the PIH health care system in Haiti.^4^

One benefit of this kind of approach is that it can promote the building of consensus among various actors, both within an organization such as PIH and across organizations, as to the planning of initiatives beyond the initial emergency response. The organizations involved can include national governmental bodies, nongovernmental organizations (NGOs), academic medical centers, universities, and corporations—all seeking to support long-term development of mental health programs in a chaotic, postdisaster setting. Given that significant challenges have been documented in the functioning of humanitarian mechanisms in Haiti both before and since the 2010 earthquake,^2^,^5^–^7^ clarity of intent and transparency in planning was, for various reasons, an essential step in moving forward effectively.

Another benefit of this approach is that it challenges the common roles and expectations of psychiatrists who wish to work in such settings, as well as broadens the scope of their activity. The psychiatrist must be prepared to assume multiple roles: as *accompagnateur*^8^,^9^ working in close proximity to the poor and to local providers, and with a long-term commitment to the challenges of everyday practice faced by the latter; as *public health program implementation manager* and innovator with skills in quality-improvement practices;^4^,^10^ as *educator* informed by the knowledge of issues in global health equity;^11^ as *multidisciplinary team–member* with an interest in supporting *task shifting* to nonspecialist providers;^10^ and as *specialized clinical consultant* with skills drawing, in particular, from collaborative primary care and consultation-liaison service models.^12^ Importantly, the psychiatrist should also act as an *integrator* of psychobiological and psychosocial perspectives on mental health and illness, working to balance approaches that strengthen social and economic structures, enhance resilience and mobilization of innate individual and community resources, and, when resources are available, address acute forms of distress and mental illness based on rational, sound clinical practice that is attuned to culture and local context.^13^–^16^

With prior experience in program implementation in Haiti, the international health care organization PIH and its Haitian sister organization Zanmi Lasante (ZL; “Partners in Health” in Haitian Creole) were among the first responders to the acute medical crisis following the earthquake. Founded in 1983, ZL delivers health care in Haiti's Artibonite Valley and Central Plateau with a staff of approximately 5000 providers, including 2500 community health workers, through 11 hospitals in partnership with the Haitian Ministry of Health (Ministère de la Santé Publique et de la Population). PIH, itself founded in Haiti in 1987 and based in Boston, has been collaborating with ZL for more than two decades. Currently working in 12 countries, PIH collaborates with NGOs and governments for the purpose of strengthening health systems, facilitating sustainable approaches, and building local capacity to deliver high quality health care.^17^ Working together, PIH and ZL have introduced novel programs to reduce transmission of, and mortality from, infectious diseases such as HIV/AIDS and tuberculosis (TB)^18^,^19^ and have established viable platforms for comprehensive health programs^20^,^21^ that are potentially conducive to the integration of dedicated mental health services.^22^

Affiliated with Harvard Medical School and a number of U.S. academic medical centers, PIH has sought to strengthen health systems in low-resource countries through four categories of action grounded in a human rights framework: the delivery of clinical and social services in community and hospital settings; the development of research based on contextual priorities; training and education in service delivery, systems management, and research methods; and advocacy regarding critical, unmet needs in health care for the poor. This case study will draw from these four categories in framing the PIH earthquake response and the building of local capacity to deliver mental health services over the long term.

## THE EMERGENCY RESPONSE: SERVICE DELIVERY

**Figure 1:**
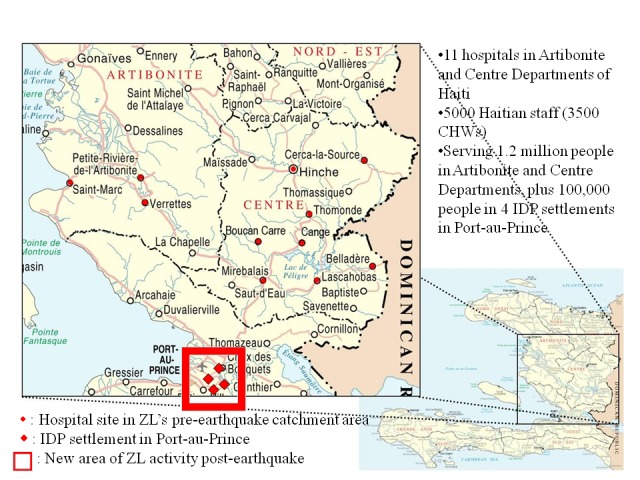
Partners in Health/Zanmi Lasante activity in post-earthquake Haiti, 2010. CHW, community health worker; IDP, internally displaced person; ZL, Zanmi Lasante.

In the acute phase of the earthquake response—in addition to covering the medical needs of 1.2 million people living in the original ZL catchment area in central Haiti—PIH/ZL took on the provision of medical care to internally displaced persons (IDPs) in four camps in Port-au-Prince with a total population of approximately 100,000 (see Figure 1). A small group within PIH/ZL, drawn from a range of disciplines and referred to below as the *Team*, took upon itself the responsibility of advocating for a sustained mental health service response.

Initial actions included assessments of existing services within and outside of the ZL catchment area, as well as meetings with Haitian mental health service leaders in Port-au-Prince, where the earthquake had caused many fatalities and the most severe physical damage. The Team worked with representatives from other organizations in the United Nations Cluster group process, meeting on a biweekly basis to discuss coordinated service strategies. Representatives of the Team met with Haiti's minister of health, facilitated by PIH's medical director. The minister acknowledged that the mental health needs of the population had been neglected prior to the earthquake and that the disaster had exposed a public sector mental health system in disrepair. People with severe mental and developmental disorders were languishing in neglected, locked inpatient wards or wandering in the streets, and few psychiatrists were working in Haiti.^23^–^25^ The minister requested PIH/ZL's support in developing a national mental health response to the disaster. The Team proposed to the Ministry of Health an initiative by which PIH/ZL would use its initial response as a foundation for a sustained, longer-term organizational commitment to developing decentralized mental health services within the ZL health care system. This initiative would serve as a model for developing a community-based mental health service structure that would be shared with the government as part of a broader planning effort organized under the leadership of the ministry.

The Team understood that building a system for mental health care in this context would require a systematic, integrated, evidence-based, multisectoral approach that prioritized local knowledge, as well as a range of cooperation and expertise larger than any single organization or institution, or the government, could provide. In the years prior to the earthquake, the ZL psychosocial program had focused primarily on the socioeconomic, educational, and psychological needs of children and families affected by HIV/AIDS or TB.^26^ Importantly, these services were buttressed by the Program on Social and Economic Rights, a system of social and economic support created by PIH/ZL to address core determinants of poor health such as lack of food, housing, or water. This preexisting medical and psychosocial platform for basic service delivery operates on the assumption that emotional distress in the context of poverty requires social and political action, as well as clinical intervention, to address preventable root causes. This platform, along with its approach, would provide a helpful launching point for the PIH/ZL post-earthquake mental health response.

In February 2010, the newly integrated PIH/ZL mental health and psychosocial Team led a series of memorial services at ZL hospital sites to commemorate both loss and survival, and to provide comfort, solace, and emotional healing to staff at ZL hospitals. The ZL psychosocial and mental health service director, a Haitian priest and psychologist, developed protocols for religious mourning services combining spiritual and psychological language. This template was adopted by the Haitian Ministry of Health and was used for national radio addresses emphasizing the importance of mourning and—explicitly—mental health. Community health workers living and working in the Port-au-Prince IDP settlements were taught to deliver these ceremonies to the population in an organized way, integrating music and communal activities, and facilitating collective grieving.

Groups identified as being especially vulnerable to mental health problems included people who sustained physical injuries and amputations, IDPs, individuals at risk of gender-based violence and children in need of protection.^27^–^32^ The earthquake's effects were also recognized to extend to people with preexisting mental disorders or significant prior histories of trauma and loss, Haitians living abroad and in the Diaspora, and health care providers and others responding to the earthquake relief effort or providing ongoing services.^33^–^38^

The Team became engaged in three primary activities: (1) finding, supporting, and treating both basic psychosocial and acute mental health needs of IDPs, (2) building capacity for overall psychosocial and mental health services, both preventive and clinical, at ZL sites, and (3) supporting the Haitian Ministry of Health in developing a national mental health plan. The Team drew from current evidence and best-practice recommendations to provide an initial mental health and psychosocial response to the emergency, consisting of (1) support to staff members. (2) launching of child- and family-specific social activities, (3) development of community programs, (4) training in psychological first aid, adapted to the Haitian context for use by Haitian psychologists and social workers, and (5) implementation of enhanced mental health services at all ZL sites, including training of primary care physicians in managing acute distress states and in using psychopharmacology. The psychopharmacologic formulary at ZL hospital sites was expanded to contain a range of options for managing acute distress states and more chronic mental illness. All of these interventions helped to stabilize individuals and communities during the early phases of the emergency.

The Team advocated internally for an increased commitment by PIH to mental health services. Within four months of the earthquake the Team obtained the resources to bring staffing numbers to 17 psychologists (up from 3) and 50 social workers and social work assistants (up from 20). These additional staff members were all Haitian nationals, and most had completed bachelor's-level training. Of the new ZL staff, 8 psychologists, 6 social workers, and 13 mental health–focused community health workers were deployed in the IDP settlements. These community health workers had themselves already been living in the IDP settlements and were selected by settlement citizen committees to help refer people suffering from acute distress responses to the clinicians working at the free-of-charge primary health clinics run by PIH/ZL. The primary care clinicians, psychologists, and pharmacists received training in psychopharmacology to prescribe medications, acting as an on-site multidisci-plinary team with the support of a Haitian psychiatrist. Initial training and supervision were provided by several visiting U.S. clinicians (psychiatrists and a psychologist), all with knowledge of the Haitian context and all drawing upon curricular materials (translated into both French and Creole) developed after the earthquake with attention to the expanded psychopharmacologic formulary at ZL. The ZL mental health clinicians also provided individual and group psychotherapy based on the training that they had received prior to the earthquake. PIH/ZL sought to replicate this basic collaborative care service model at all ZL hospital sites, adapted to the skill sets of those working in community health, primary care, and mental health consultation-liaison capacities, with one psychiatrist serving the entire system. Each ZL hospital site was assigned one psychologist, whose responsibilities were broadened to encompass both psychosocial services related to HIV/TB and management of clinically severe presentations, including trauma-related symptoms and chronic mental disorders. The psychologists were also charged with assembling multidisciplinary teams at each hospital site in the Central Plateau and Artibonite, with each team to include at least one physician for prescribing medications. At one-and-a-half years after the earthquake, ZL staff had documented 20,000 individual and group appointments for mental health and psychosocial needs in the IDP settlements and at ZL hospital sites.

In addition to their clinical work, the IDP settlement teams organized memorial services, games for children, community educational and psychosocial support activities, and psychoeducational meetings. Aware that more than a quarter of the population living in the IDP settlements were children under five years of age, PIH/ZL expanded child protection efforts by creating safe spaces for children and actively reporting unaccompanied children to a national referral center run by UNICEF. Within the ZL catchment area, special efforts were also made to embed psychological support within a new rehabilitation program for amputees. As rehabilitation teams followed patients into the community upon hospital discharge, they increasingly came into contact with people with preexisting mental disorders and disabilities requiring intervention.

Ten months after the earthquake, a cholera outbreak hit an already devastated Haiti, causing widespread suffering, death, and fear. Those contracting the illness were stigmatized, as the illness's precipitously rapid lethality was compounded by a shortage of access to health services, inadequate water supply, and lack of sanitation, particularly in rural areas. The Team was once again mobilized to provide memorial services—and, as had occurred after the earthquake, on short notice because of the need for immediate burial. These ceremonies, adapted for the families of patients who died unexpectedly, were designed to help them express their grief in an environment ripe with fear of a disease that had been absent from the Haitian context for more than a century. The Team also developed a support group curriculum for cholera survivors to assist them in reintegrating into their families and regaining self-confidence and a positive body image. These groups were adapted from a family-focused, psychosocial group intervention for children with HIV and their caregivers that ZL had been utilizing at hospital and clinic sites prior to the earthquake. Unexpectedly, the need for these groups became increasingly urgent as patients were being abandoned at treatment centers or refused entry to their homes due to fear that they would spread the disease.

## STRENGTHENING THE HEALTH SYSTEM FOR THE LONG TERM

PIH has worked to strengthen and expand the delivery of high-quality, comprehensive health care to the poor in countries throughout the world in collaboration with government ministries of health. The integration of research, training, education, and advocacy, along with their linkage to service delivery, has been central to advancing this core mission. With regard to mental health care, the Team recognized that the need for safe, effective, and culturally sound services in the aftermath of the Haiti earthquake would require an innovative approach that capitalized on the existing strengths of the PIH/ZL system. Also, while managing the emergency response in the first few weeks after the earthquake, the Team was carefully assessing the substantial progress that had been made globally in research on treatment dissemination to address mental disorders in low-income regions.

With increasing recognition of the burden of mental disorders globally,^39^–^47^ there has been a growing consensus regarding both basic *treatment packages*^48^,^49^ for common mental health conditions and the utility of *task shifting*^22^, ^50^–^55^—that is, using nonspecialists, such as primary care physicians and community health workers, to carry out the elements of these packages—as a key strategy for closing the enormous treatment gaps that exist in low-resource contexts. Mixed qualitative and quantitative research methods have also contributed to our understanding of how to use and adapt this emerging baseline consensus to meet local conceptions of mental health and illness. It was from these elements—agreement on good treatment, on the range of skills useful for adoption by nonspecialist providers, and on methods for adapting clinical tools and care-pathway design—that the team developed an implementation model to guide expansion of community-based mental health services. This model, which capitalized on the strengths of the ZL community health worker network, was designed to facilitate the scaling up of community-based mental health services for the long-term—first by linking all eleven ZL hospital sites and then by potentially expanding through the sharing of successes with other organizations and the Haitian Ministry of Health.

Two sets of discussions—one among colleagues involved in planning a framework for integration of mental health into primary care as part of the Millennium Villages Project in sub-Saharan Africa, and one among members of a consortium that included Haiti-based institutions (PIH/ZL, Haitian Ministry of Health, and Interuniversity Institute for Research and Development) and U.S. academic medical centers (Program in Global Mental Health and Social Change at Harvard Medical School, and Program in Global Mental Health at New York University School of Medicine)—led to a systematic, collaborative process that facilitated the articulation of a scalable model for mental health service delivery within the ZL system called *5* × *5*.^4^ In this stepwise process, structured, qualitative information on beliefs, practices, and local priorities about mental health conditions are used to develop a best practice–based template.

### Research

**Figure 2:**
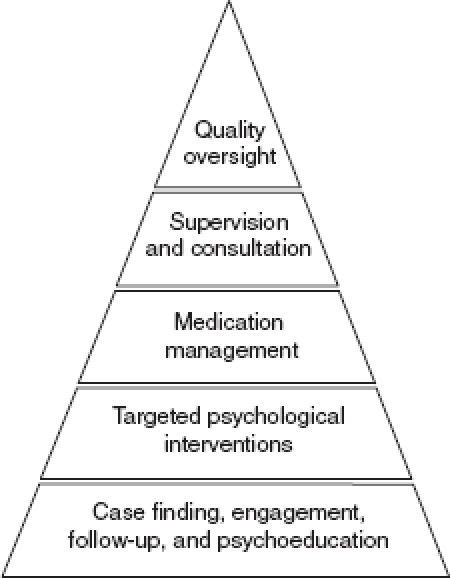
Key skill packages of the 5 × 5 Model. Adapted with permission from Belkin G et al. (2011).^4^

The 5 × 5 Model identifies five key skill types or *skill packages* that provide a mental health service—specific platform to manage accepted, evidence-based, care-pathway algorithms for common disorders. These packages are consistent with broad target conditions outlined in the World Health Organization (WHO) mental health intervention guide for mental, neurological, and substance use disorders in non-specialist health settings, which was recently developed by the WHO Mental Health Gap Action Programme (see Figure 2).^56^,^57^

**Figure 3:**
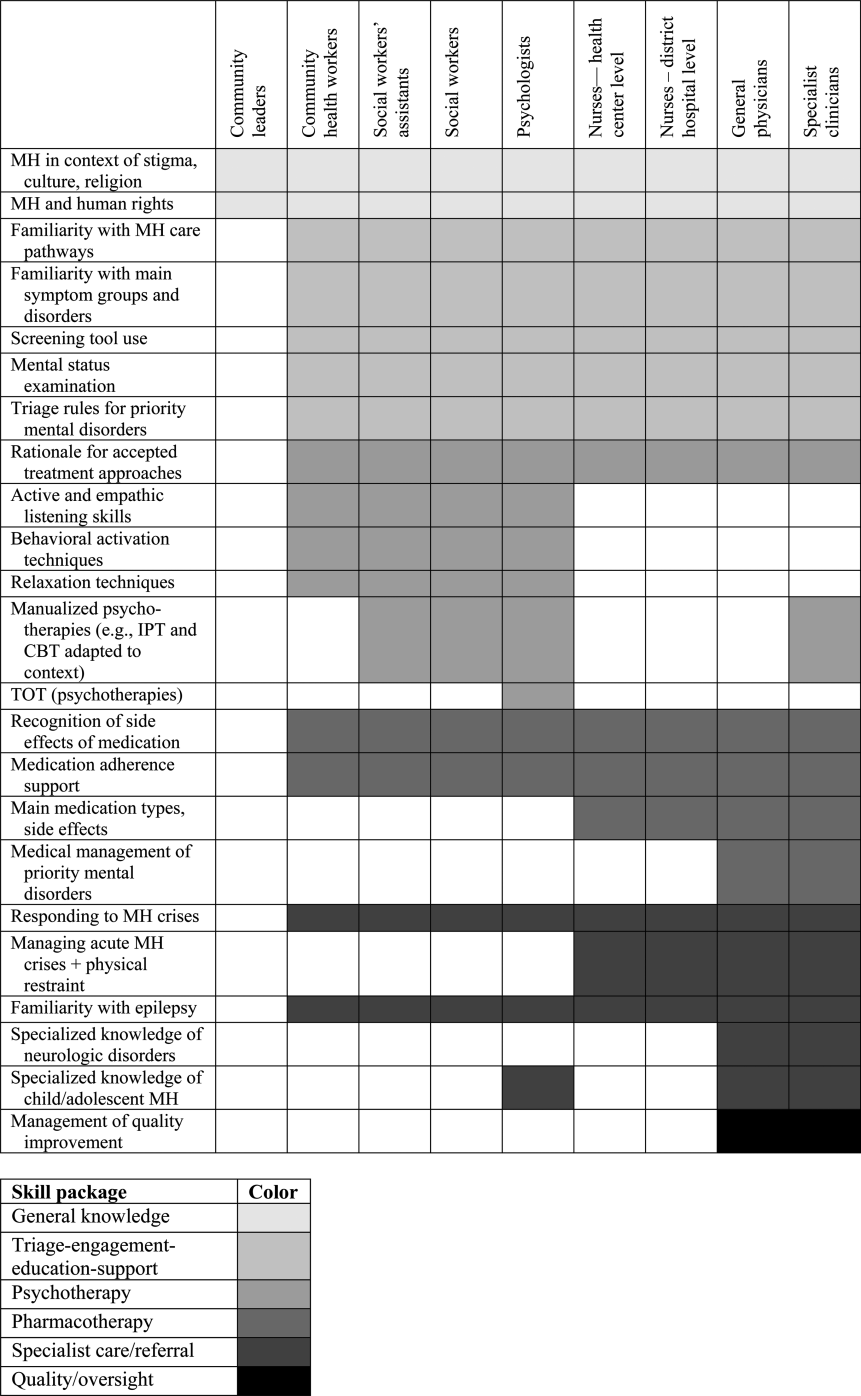
Mapping skill areas to provider roles as part of the development process for the care pathway. CBT, cognitive-behavioral therapy; IPT, interpersonal psychotherapy; MH, mental health; TOT, Training of Trainers.

The five skill packages are linked by five sequential *implementation rules* (hence, 5 × 5): (1) assess context first, (2) identify priority care pathways, (3) specify decision-support tools, supervision, and triage rules, (4) use quality-improvement practices, and (5) plan for sustainability and capacity building. The core skill-types are matched to particular health care provider roles specific to the health care system in question and are further adapted to the local context (see Figure 3).

**Figure 4:**
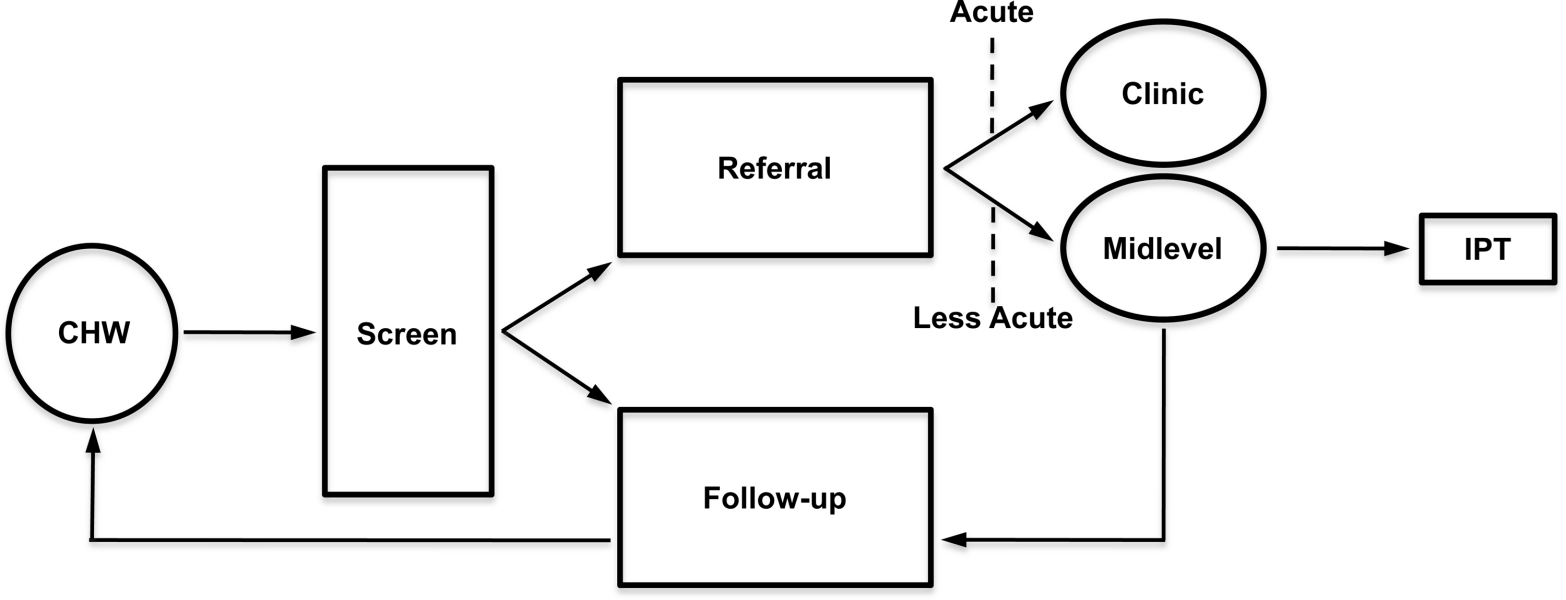
Basic care pathway for depression, assigning skill sets to specific human resources (i.e., task shifting). Services for acute and severe depressive disorders are provided by psychologists and physicians following screening and referral from CHWs. Mild to moderate depression will be treated by midlevel providers (social workers and social work assistants) trained in IPT. CHW, community health worker; IPT, interpersonal psychotherapy.

With the technical support of the other consortium members, PIH/ZL has completed the first three steps defined by the implementation rules: (1) a qualitative assessment of local beliefs and perceived needs in the community, completed in partnership with the Interuniversity Institute for Research and Development; (2) validation of a symptom-scale tool for use by community health workers to track problems related specifically to depression; and (3) the assignment of different skill sets to specific human resources, supported by a curriculum integrating accepted treatment guideline steps for depression care (see Figure 4). As part of this curriculum, PIH/ZL has begun to adapt to the Haitian context manualized treatments, such as interpersonal psychotherapy, shown to be suitable for use by trained laypersons in low-income settings.^58^–^61^ The Haitian Ministry of Health leadership has been engaged at each step and has agreed to collaborate in a series of workshops with key government representatives and district officers. The workshops will also include representatives from other NGOs working in Haiti that wish to participate in adopting the model. The consortium has sought to promote shared learning among the participants (ZL, the Haitian Ministry of Health, and other Haitian NGOs) with the goal of maximizing the potential reach of the 5 × 5 Model within Haiti—if its feasibility is established within the ZL health care system.

Following on the initial steps to articulate a care pathway for depression at a small number of pilot sites (Cange, Mirebalais, and Petite Rivière), the model will be extended to other sites. With the necessary functionality in place, new care pathways for other mental disorders will be added sequentially over time, using repeated cycles of the prior steps listed above. This process will include adapting the relevant care-pathway components to the work routine of community health workers, nurses, midlevel providers (such as social workers and social work assistants), and more specialized clinicians (such as psychologists and physicians). Appropriate information-management infrastructure and technology holds the potential to enhance the process of expanding tiered, health worker–based, integrated care pathways. Point-of-care, handheld cellphone–based treatment algorithms can extend the clinics' points of contact into the community, allowing for real-time gathering of information and the ability to provide feedback and quality supervision. It is also hoped that more sophisticated qualitative study will ensure over time that the evolving system of care is consistently reflective of local and regional needs, perceptions, and realities.

Initial funding for the systems-building proposal was obtained from several foundations through active engagement with organizations expressing interest in post-earthquake mental health services. In a 2006–08 U.S. National Institute of Mental Health (NIMH)-funded mental health services research project that evaluated a family-focused, psychosocial group intervention for children with HIV and their caregivers at ZL sites, significant reductions were found in the depressive symptoms of caregivers and in the psychological symptoms of youth.^26^ In the years immediately preceding the earthquake, these positive results led to ZL's retaining its three clinical psychologists. Within a year after the earthquake, several other research proposals were developed. One resulting NIMH award (in 2011) is for a feasibility study to develop research capacity through ZL—in particular, to develop a school-based mental health intervention for youth in Haiti's Central Plateau^62^ in collaboration with Harvard Medical School.

### Training and Education

The Team recognized that not only in Haiti but also more broadly, health care providers, trainees, and students need to be educated in implementing multidisciplinary systems of mental health care. Moreover, collaborative sharing of experiences across nationalities, cultures, and disciplines is important in ensuring that programs in low-resource (and often postcolonial) settings are not imposed from outside but driven by local needs and leaders. Training that is “bidirectional” in time and resource allocation, as well as in spirit, became a key aspect of the Team's vision for education in mental health service delivery, both for Haitian and U.S. clinicians and trainees.

One and a half years after the earthquake, PIH hired a full-time psychiatrist, the Dr. Mario Pagenel Fellow in Global Mental Health Delivery, to train and clinically support expert ZL mental health teams. Cosponsored by Harvard Medical School, the fellowship honors the life and work of Dr. Mario Pagenel, director of training and medical education at ZL, who had a special interest in mental health and health equity, and who had died in the earthquake. The fellowship provides U.S. psychiatrists and graduating senior psychiatric residents with the opportunity to develop service and academic career interests in global mental health—in particular, by supporting the development of mental health training programs for clinicians at PIH sites in Haiti and Rwanda. For one month, prior to moving to the sites, the fellow participates in the Harvard University Global Health Effectiveness Program. This program trains health care professionals in topics related to health program management in low-resource settings. In Haiti, the fellow works side by side with ZL clinicians who are the primary providers of services, accompanying them as they implement clinical mental health interventions and generally supporting the evolving service model. The fellow is also mentored in developing an area of academic focus in global health–related research or education. Simultaneously, the program aims to create opportunities for Haitian health care providers to develop careers as mental health practitioners, to distinguish themselves as leaders, and to promote their desire to continue to practice in their home communities.

### Advocacy

Thanks to PIH/ZL's increased service commitment, a number of patients—whether injured in the earthquake, suffering from cholera, or with long-standing mental illness— received care that helped them reintegrate into meaningful activities such as church, choir, and school. The improvement of their social functioning noticeably reduced stigma and changed perceptions, both in the community and within the medical system. October 10, 2010, marked the first time that PIH/ZL observed World Mental Health Day. Throughout the entire month, ZL psychologists and social workers organized mass education and cultural events, including contests and other activities at schools, churches, religious meetings, and sporting events. The purpose was to educate communities on the importance of promoting both physical and mental health, to reduce the stigma associated with mental disorders, and to inform the population of the free-of-charge mental health services available at all ZL hospitals. Leading Haitian psychiatrists, the Ministry of Health, and ZL collaborated to create a theme for World Mental Health Day, and agreed on a logo meant both to raise awareness and to circumvent stigma. In 2011, the Haitian Ministry of Health and PIH/ZL observed World Mental Health Day for the second consecutive year under the banner: “With a clear mind, your body is stronger (Ak tèt klè kò a pi djanm).”

## CONCLUSIONS

Mental health and psychosocial service responses following disasters in low-resource contexts have been beset by difficulties in coordinating the interventions by NGOs, groups, and individuals with the priorities of governments and local communities, especially in the implementation of culturally relevant practices. This case study has described efforts to harness the current movement in global mental health to strengthen the Haitian public sector in the earthquake's aftermath. These efforts have involved (1) the delivery of disaster response services relevant to the Haitian context and led by local practitioners following the earthquake, (2) the development and implementation of a planning framework (the 5 × 5 Model) to guide the activities of PIH/ZL over the longer term, (3) the coordination of PIH/ZL with multiple organizations, including the Haitian government, in mental health service planning and delivery, and (4) the linkage of research, training, and advocacy activities to the services delivered by PIH/ZL in collaboration with U.S. academic medical centers. While this case study describes efforts especially pertinent to a low-income country responding to a natural disaster, change-management processes such as that outlined by the 5 × 5 Model are also relevant to mental health service planning in middle- and high-income countries.^63^ This case also describes additional roles for psychiatrists in situations where mental health services have been especially neglected and stigmatized.

There is room to be hopeful that this and similar efforts will stimulate a broader dialogue on the need to integrate models of community-based mental health care in Haiti. In June 2011, a number of organizations, including PIH/ZL, met under the leadership of the Ministry of Health, the World Health Organization, and the Pan-American Health Organization to continue formal planning on a shared strategy for a national mental health plan. It is hoped that this process will gain momentum and become a sustained commitment to the building of long-term solutions.

The authors thank Helen Knight for help with Figures 3 and 4, Andrew Rasmussen, PhD, for developing the depression screening instrument, and Helen Verdeli, PhD, for the contextual adaptation of empirically based psychotherapies and skills training of health care providers. This work was made possible by the ongoing support of the leadership and 18. staff of PIH and ZL.
